# Simultaneous silencing of TGF-β1 and COX-2 reduces human skin hypertrophic scar through activation of fibroblast apoptosis

**DOI:** 10.18632/oncotarget.20869

**Published:** 2017-09-14

**Authors:** Jia Zhou, Yixuan Zhao, Vera Simonenko, John J. Xu, Kai Liu, Deling Wang, Jingli Shi, Tianyi Zhong, Lixia Zhang, Lun Zeng, Bin Huang, Shenggao Tang, Alan Y. Lu, A. James Mixson, Yangbai Sun, Patrick Y. Lu, Qingfeng Li

**Affiliations:** ^1^ Shanghai Ninth People's Hospital, Shanghai Jiao Tong University School of Medicine, Shanghai, China; ^2^ Sirnaomics, Inc., Gaithersburg, MD, USA; ^3^ Suzhou Sirnaomics Pharmaceuticals, Ltd., Biobay, Suzhou, China; ^4^ Guangzhou Xiangxue Pharmaceuticals, Co. Ltd., Guangzhou, China; ^5^ Guangzhou Nanotides Pharmaceuticals, Co. Ltd., Guangzhou, China; ^6^ Department of Pathology, School of Medicine, University of Maryland, Baltimore, MD, USA

**Keywords:** siRNA therapeutics, hypertrophic scar, TGF-β1, COX-2, synergistic effect

## Abstract

Excessive skin scars due to elective operations or trauma represent a challenging clinical problem. Pathophysiology of hypertrophic scars entails a prolonged inflammatory and proliferative phase of wound healing. Over expression of TGF-β1 and COX-2 play key regulatory roles of the aberrant fibrogenic responses and proinflammatory mediators. When we silenced TGF-β1 and COX-2 expression simultaneously in primary human fibroblasts, a marked increase in the apoptotic cell population occurred in contrast to those only treated with either TGF-β1 or COX-2 siRNA alone. Furthermore, using human hypertrophic scar and skin graft implant models in mice, we observed significant size reductions of the implanted tissues following intra-scar administration of TGF-β1/COX-2 specific siRNA combination packaged with Histidine Lysine Polymer (HKP). Gene expression analyses of those treated tissues revealed silencing of the target gene along with down regulations of pro-fibrotic factors such as α-SMA, hydroxyproline acid, Collagen 1 and Collagen 3. Using TUNEL assay detection, we found that the human fibroblasts in the implanted tissues treated with the TGF-β1/COX-2_siRNAs_ combination exhibited significant apoptotic activity. Therefore we conclude that a synergistic effect of the TGF-β1/COX-2siRNAs combination contributed to the size reductions of the hypertrophic scar implants, through activation of fibroblast apoptosis and re-balancing between scar tissue deposition and degradation.

## INTRODUCTION

Human hypertrophic scar reduction and management are major therapeutic challenges due to lack of in-depth understanding of the underlying mechanism and the few validated treatment strategies available [1]. Understanding the pathophysiology of fibrosis may lead to a novel therapeutic with improved clinical benefit [2]. Fibrosis is defined by excessive accumulation of extracellular matrix (ECM) in and around the damaged tissue, which can lead to permanent scarring [3]. Hypertrophic scar (HS) is the result of a disrupted balance between ECM protein deposition and degradation during the dermal wound healing process [4]. It is characterized by the prolonged inflammatory response to injury resulting in an increased vascularization, hypercellularity and excessive collagen deposition from local fibroblasts [5]. Fibroblasts are the most common cells in connective tissue, playing a key role in the wound healing process and can differentiate into myofibroblasts that results in increased ECM synthesis and tissue contraction [6, 7].

HS fibroblasts (HSFs) have demonstrated reduced collagenase expression [8], but elevated expression of TGF-β1, which lead to an excessive deposition of collagen resulting in HS formation [9, 10]. HS myofibroblasts are less sensitive to apoptotic signals but exhibit high expression levels of α-smooth muscle actin (α-SMA). Abundant evidence has indisputably confirmed the central role of TGF-β1 in driving fibrosis and HS, especially when secreted from macrophages and fibroblasts [11]. In contrast, the role of prostaglandin E2 (PGE2) synthesized by cyclooxygenases (COX-2) during wound healing, is highly controversial. Although *in vitro* studies have provided some evidence to support a negative feedback loop, in that high levels of COX-2 and PGE2 expressions can inhibit TGF-β1-mediated fibrogenesis activities in various cell types, it has been suggested that these observations are due to the terminal stages of the cells used and do not reflect the early responses of TGF-β1 and COX-2 pathways to the injury *in vivo* [12]. In addition, animal studies that demonstrated lower PGE2 and COX2 expression associated with reduced collagen synthesis in scarless fetal wounds [13] suggests that COX-2 could be a potential therapeutic target for limiting scar formation [14].

Studies with many treatment modalities for excessive scarring have not achieved satisfying remission. Those treatments included surgical excision, radiation, corticosteroid injections, cryotherapy, laser vaporization, topical 5-fluorouracil, bleomycin injection, paper tape to eliminate scar tension, pressure garment therapy, silicone gel sheeting, and short term use of ozonated oil. Several novel therapeutic modalities are also in development including TGF-β1 antagonists, exogenous PGE2, stem cell therapy and antisense to Connective Tissue Growth Factor (CTGF), encouraged by emerging preliminary findings in both animal models and human studies, EXC001 [15] and RXI109 [16]. To determine whether simultaneous targeting of TGF-β1 and COX2 could improve outcomes in HS, we designed two siRNA duplexes specific to TGF-β1 and COX-2 mRNA sequences and tested them in human fibroblasts and human HS tissue implants mouse models.

Efficient siRNA delivery into skin wound site or HS tissue is required to achieve a significant therapeutic effect, and at the same time, minimize “off-target” and other immune stimulation side effects. One previous approach we developed is using a biodegradable polypeptide molecule, Histidine-Lysine co-Polymer (HKP), to package siRNA oligonucleotides into nanoparticles for enhancing siRNA delivery *in vivo* [17, 18]. To improve intradermal administration, we developed a process to formulate HKP with the selected siRNA duplexes targeting both TGF-β1 and COX-2 into a nanoparticle aqueous suspension. Intradermal injection of the HKP-siRNA nanoparticle resulted in a synergistic reduction in the size of the hypertrophic scar. Here we provide the first report that a novel dual-targeted siRNA therapeutic approach exhibits potent anti-fibrotic activity with a newly discovered mechanism of action.

## RESULTS

### Simultaneous silencing of TGF-β1 and COX-2 induces human fibroblast apoptosis *in vitro*

**Figure 1:**
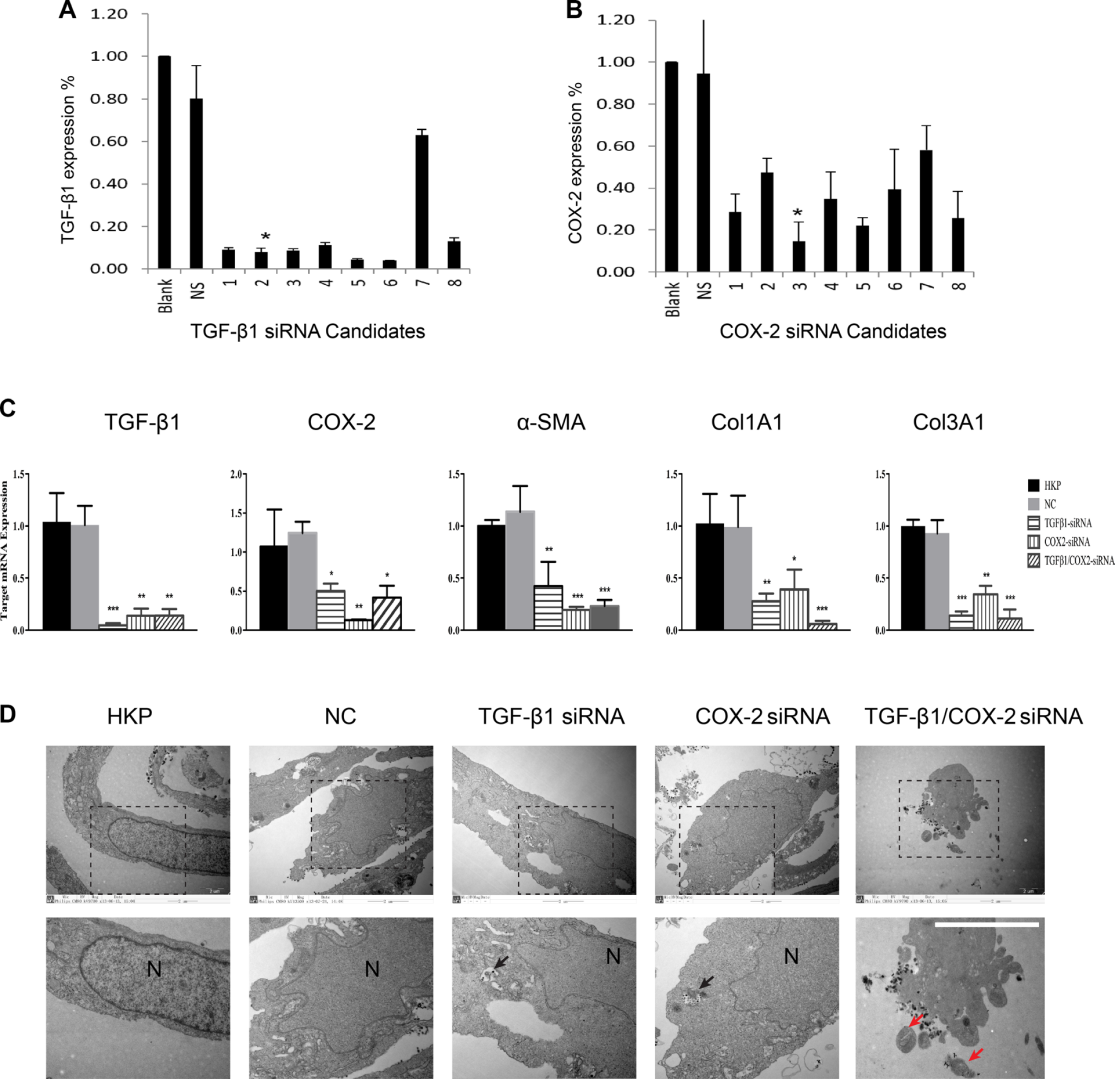
Selections of TGF-β1 and COX-2 Specific siRNAs (**A**) siRNA selection for targeting TGF-β1 *in vitro*. In silico selected 8 siRNA duplexes were transfected into human prostate cancer cell PC-3 to determine their silencing efficacy. After transfection, total RNA was isolated and qRT-PCR analysis was performed. The control siRNA is NS (Lu25-a 5′-r(GAGGAGCCUUCAGGAUUACAAGAUU)-3′ 5′-r(AAUCUUGUAAUCCUGAAGGC-UCCUC)-3′: 2 μg). The star represents the optimal silencing activity of the selected siRNA duplex, *N* = 3. (**B**) siRNA selection for targeting COX-2 *in vitro*. In silico selected 8 siRNA duplexes were transfected into human prostate cancer cell PC-3 to determine their silencing efficacy, followed by total RNA isolation and qRT-PCR analysis. The star indicates the optimal silencing activity by the selected siRNA duplexes, *N* = 3. (**C**) Comparisons of target gene silencing. mRNA levels of TGF-β1, COX-2, a-SMA, Col1A1 and Col3A1 from human hypertrophic scar fibroblasts (HSFs), after their transfection with either TGF-β1siRNA, or COX-2siRNA, or TGF-β1/COX-2siRNAs (5 ug/ml). NC: Negative control with non-targeting siRNA. HKP: Vehicle control without scrambled siRNA, (HKP alone). The summary data are from three independent experiments. **P < 0.01, ***P < 0.001. (**D**) Electron microscope images of the fibroblast cells transfected with the TGF-β1/COX-2siRNAs illustrates apoptotic activity, where N indicates nucleus, black arrows indicate HKP-siRNA particles and red arrows indicate apoptosis bodies.

We first designed siRNA sequences specific to human TGF-β1 and COX-2 mRNAs in silico and then tested the efficacy of these sequences based on cell transfection and analysis using qRT-PCR (Tables 1–2). The siRNAs were selected based on their silencing efficiencies and toxicity profiles affecting cells (Figure 1A–1B). When human fibroblasts isolated from the hypertrophic scar tissue were transfected with the selected siRNAs targeting either TGF-β1 or COX-2 individually or in combination, we observed an efficient siRNA entry (Supplementary Figure 1) into the cells at different stages, from initial endocytosis to endosome release of the siRNAs. The measurements of target gene silencing after the transfection indicated significant knockdown of target gene expression, with either TGF-β1siRNA or COX-2siRNA themselves, or upon combination TGF-β1/COX-2siRNAs. Interestingly, not only TGF-β1siRNA and TGF-β1/COX-2siRNAs combination were able to silence TGF-β1 expression, the COX-2siRNA was also able to down regulate TGF-β1 expression. This result suggests a potential interconnection between TGF-β1 and COX-2 pathways. Similarly, TGF-β1siRNA was also able to silence COX-2 expression significantly. As the target genes were silenced, other pro-fibrotic factors such as α-SMA, Collagen 1 (Col1A1) and Collagen 3 (Col3A1) were also down regulated in the cells (Figure 1C). Cells treated with HKP alone and with NC siRNAs served as control. These results indicate that silencing TGF-β1 and COX-2 simultaneously in the fibroblasts down regulated multiple pro-fibrotic factors.

**Table 1:** siRNA sequences used in the study

	Sense	Antisense
**Lu25-a**	5′-r(GAGGAGCCUUCAGGAUUACAAGAUU)-3′	5′-r(AAUCUUGUAAUCCUGAAGGCUCCUC)-3′
**GFP-1**	5′-r(GCUGACCCUGAAGUUCAUCUGCAUU)-3′	5′-r(AAUGCAGAUGAACUUCAGGGUCAGC)-3′
**hmTF-25-1**	5′-r(GGAUCCACGAGCCCAAGGGCUACCA)-3′	5′-r(UGGUAGCCCUUGGGCUCGUGGAUCC)-3′
**hmTF-25-2**	5′-r(GAGCACCAUUCUCCUUGAAAGGACU)-3′	5′-r(AGUCCUUUCAAGGAGAAUGGUGCUC)-3′
**hmTF-25-3**	5′-r(CCUCAAUUCAGUCUCUCAUCUGCAA)-3′	5′-r(UUGCAGAUGAGAGACUGAAUUGAGG)-3′
**hmTF-25-4**	5′-r(GAUCCACGAGCCCAAGGGCUACCAU)-3′	5′-r(AUGGUAGCCCUUGGGCUCGUGGAUC)-3′
**hmTF-25-5**	5′-r(CACGAGCCCAAGGGCUACCAUGCCA)-3′	5′-r(UGGCAUGGUAGCCCUUGGGCUCGUG)-3′
**hmTF-25-6**	5′-r(GAGGUCACCCGCGUGCUAAUGGUGG)-3′	5′-r(CCACCAUUAGCACGCGGGUGACCUC)-3′
**hmTF-25-7**	5′-r(GUACAACAGCACCCGCGACCGGGUG)-3′	5′-r(CACCCGGUCGCGGGUGCUGUUGUAC)-3′
**hmTF-25-8**	5′-r(GUGGAUCCACGAGCCCAAGGGCUAC)-3′	5′-r(GUAGCCCUUGGGCUCGUGGAUCCAC)-3′
**hmCX-25-1**	5′-r(GGUCUGGUGCCUGGUCUGAUGAUGU)-3′	5′-r(ACAUCAUCAGACCAGGCACCAGACC)-3′
**hmCX-25-2**	5′-r(GAGCACCAUUCUCCUUGAAAGGACU)-3′	5′-r(AGUCCUUUCAAGGAGAAUGGUGCUC)-3′
**hmCX-25-3**	5′-r(CCUCAAUUCAGUCUCUCAUCUGCAA)-3′	5′-r(UUGCAGAUGAGAGACUGAAUUGAGG)-3′
**hmCX-25-4**	5′-r(GAUGUUUGCAUUCUUUGCCCAGCAC)-3′	5′-r(GUGCUGGGCAAAGAAUGCAAACAUC)-3′
**hmCX-25-5**	5′-r(GUCUUUGGUCUGGUGCCUGGUCUGA)-3′	5′-r(UCAGACCAGGCACCAGACCAAAGAC)-3′
**hmCX-25-6**	5′-r(GUGCCUGGUCUGAUGAUGUAUGCCA)-3′	5′-r(UGGCAUACAUCAUCAGACCAGGCAC)-3
**hmCX-25-7**	5′-r(CACCAUUCUCCUUGAAAGGACUUAU)-3′	5′-r(AUAAGUCCUUUCAAGGAGAAUGGUG)-3′
**hmCX-25-8**	5′-r(CAAUUCAGUCUCUCAUCUGCAAUAA)-3′	5′-r(UUAUUGCAGAUGAGAGACUGAAUUG)-3′

Lu25: siRNA targeting Luciferase; GFP-1: siRNA targeting Green Fluorescence Protein; hmTF (X): siRNAs targeting human TGF-beta1; huCX (X): siRNAs targeting human COX-2.

**Table 2:** Samples information used in the study

No.	Sex	Age	Biopsy site	Duration time(months)
**HS1**	M	3	Foot	12
**HS2**	F	3	Neck	6
**HS3**	M	20	Jaw	12
**NS1**	M	3	Belly	
**NS2**	F	3	Thigh	
**NS3**	M	20	Neck	

HS: Hypertrophic scar; NS: Normal skin; M: Male; F: Female.

**Figure 2:**
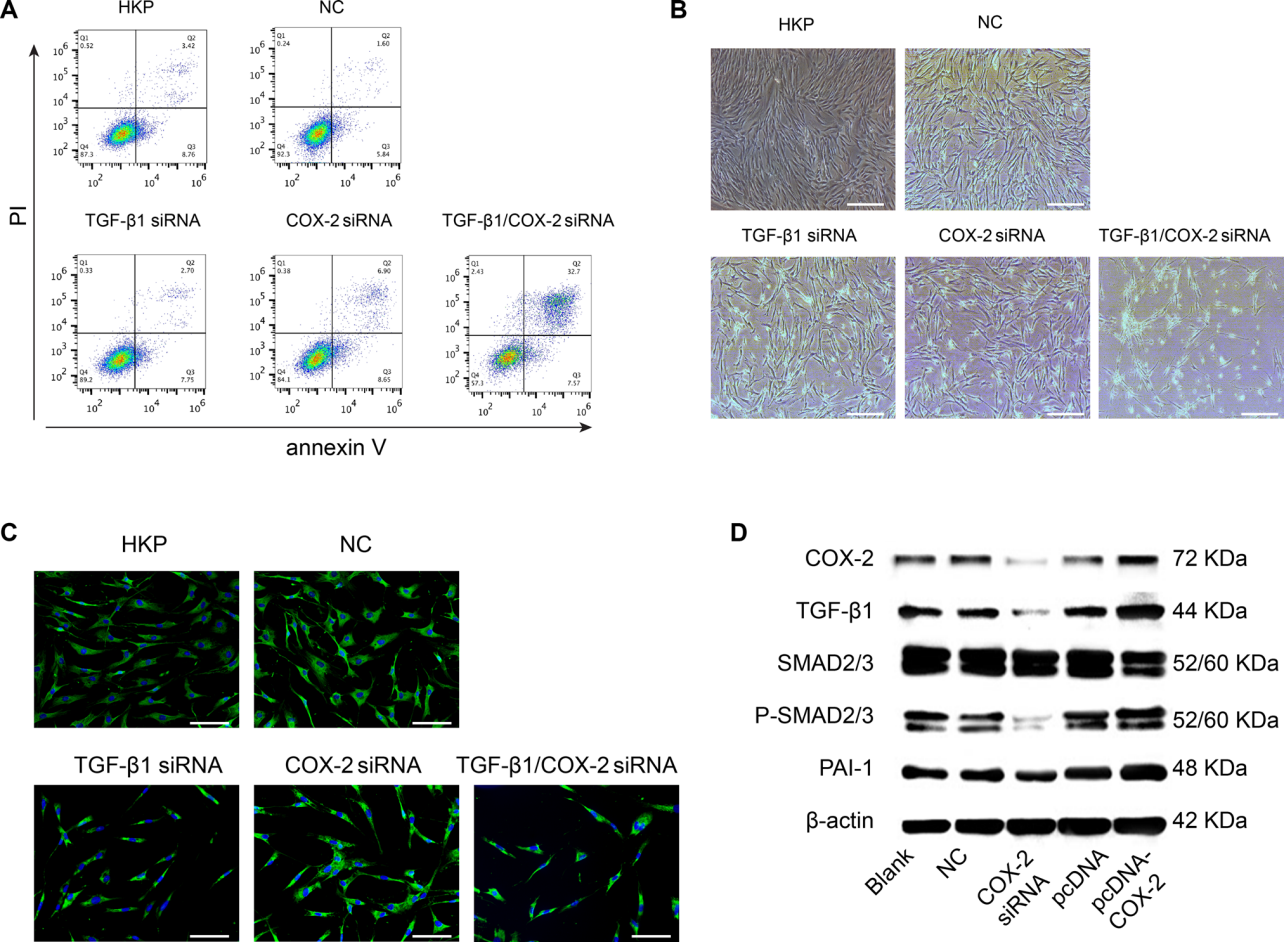
Phenotypical Effects after Target Genes Silenced by TGF-β1 and COX-2 Specific siRNA Duplexes (**A**) Apoptotic activity of the human fibroblasts was induced when TGF-β1 and COX-2 were silenced simultaneously. The lower right panel illustrated significant shift of the apoptotic cell population. HSFs in the absence or presence of non-targeting siRNAs loaded by HKP served as control. A representative result of three independent experiments is shown. (**B**) Morphological changes of the fibroblasts after the siRNA transfection. The lower right panel shows lower density of the fibroblasts with slimmer cell shape. (**C**) The a-SMA protein expression within the cells was significantly down regulated as shown with green dye labeled mAb against a-SMA, with the nucleus counterstained with DAPI. (**D**) After HSFs were transfected with siRNA-COX2, there were marked decreases in protein expression levels of COX2, TGF-β1, SMAD2/3, p-SMAD2/3 and PAI-1; conversely, these proteins were up-regulated after transfection of these cells with pcDNA3.1-COX2.

We further investigated the fate of the fibroblasts when those pro-fibrotic factors were down regulated. Electron microscope images (Figure 1D) of the fibroblast cells transfected with the either TGF-β1siRNA or COX-2siRNA only, or TGF-β1/COX-2siRNAs combination, illustrated that the combination treatment induced apoptosis in fibroblasts, but apoptosis was not induced with individual siRNA treatments. FACS analyses of the HSFs treated with the TGF-β1/COX-2siRNAs combination revealed a marked increase in the apoptotic cell population (Figure 2A), compared to those treated with either TGF-β1siRNA or COX-2siRNA individually. Fibroblasts treated with the TGF-β1/COX-2siRNAs combination showed much lower cell density and a different morphology (narrow cell shape (Figure 2B)). When we examined the expression of α-SMA protein in the human fibroblasts after the TGF-β1/COX-2siRNAs combination treatment, a significant reduction was observed compared to the individual siRNA treatments as measured by immunofluorescence staining (Figure 2C). Inhibitors of TGFβ receptor type I (TBR1) and COX2 were also used to validate the effects of abrogating TGF-β1 and COX2 function which down-regulated fibrotic factors expression and promoted cell apoptosis on HSFs (Supplementary Figure 2). To explore the role of COX2 in modulating the activation of TGFβ signaling pathway, we used COX2-siRNA and pcDNA3.1-COX2 over expression vector respectively. When transfected with COX2-siRNA, we observed a decreased expression level of canonical TGFβ signaling pathway downstream proteins, SMAD2/3, phosphorylated SMAD2/3(indicative of active TGFβ signaling) and PAI-1 (TGF-β1 target gene) by Western blot; this verified that COX2 modulated the TGFβ signaling pathway (Figure 2D). We also measured the level of hydroxyproline acid (HPC) in cell culture, an indicator of the synthetic activity of ECM proteins and a hallmark of tissue fibrosis, using human fibroblasts treated with TGF-β1/COX-2siRNAs combination (Supplementary Figure 3). Together, the results clearly indicate that when TGF-β1 and COX-2 gene expressions in the human fibroblasts were silenced simultaneously, down regulations of multiple fibrotic factors occurred. Consequently, the treated fibroblasts became less active and more apoptotic.

### HKP enhances siRNA delivery into human hypertrophic scar

**Figure 3:**
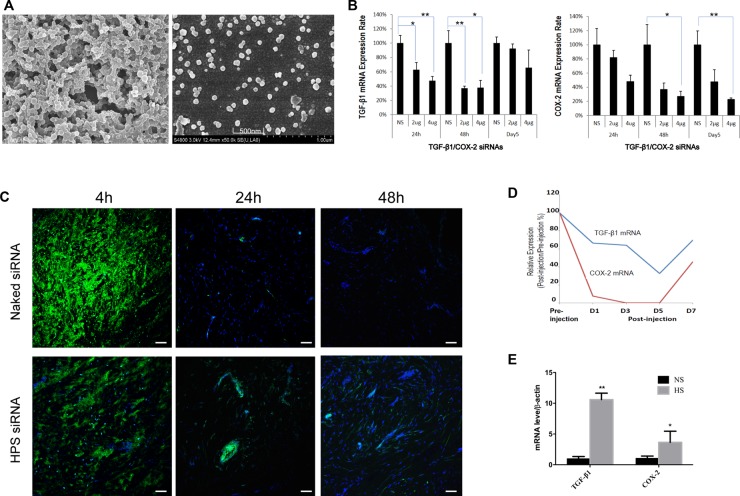
HKP Enhances Intra-scar Delivery of siRNA (**A**) SEM image of HKP (TGF-β1/COX-2siRNAs) nanoparticles. After resuspension of lyophilized HKP (siRNA) nanoparticles in aqueous solution, these particles had an average size of 150 nm in size and similar size distribution, properties of which are typical for intra-scar siRNA administration. (**B**) Real-Time qPCR analysis of the tissue samples revealed HKP packaged TGF-β1/COX-2siRNAs knocking down TGF-β1 and COX-2 in a dose-dependent manner. Whereas TGF-β1 was down regulated more between 24–48 hours post treatment, COX-2 decreased more between 48 and 96 hours post treatment (*n* = 6). NS is Lu25-a 5′-r(GAGGAGCCUUCAGGAUUACAAGAUU)-3′ 5′-r(AAUCUUGUAAUCCUGAAGGCUCCUC)-3′, at 2μg serving as control siRNA. (**C**) The siRNA-Alexa Fluor labeled and HKP-packaged siRNA-Alexa Fluor labeled were compared *in vivo* for their duration and dispersion after local intra-scar injection, using human hypertrophic scar tissue implant model with mice, at three time points: 0 hour, 24 hour and 48 hour post administration. HKP formulated siRNA resulted in a prolonged siRNA duration after intrascar injection into human hypertrophic scar implant. (**D**) Injections of HKP (TGF-β1/COX-2siRNAS) nanoparticle solution into the human hypertrophic scar resulted in down regulations of TGF-β1 and COX-2 expressions in the tissue, up to 5 days, based on the qRT-PCR analyses. (**E**) Comparison of TGF-β1 and COX-2 expressions in human normal skin tissue (NS) and human hypertrophic scar tissue (HS) (*n* = 3, *P < 0.05).

To ensure efficient siRNA delivery to the hypertrophic scar, we selected a biodegradable histidine-lysine polypeptides (HKP) that has been demonstrated to provide efficient siRNA delivery *in vivo* [17, 18]. When HKP and siRNA are mixed in aqueous solution with an optimized N/P ratio (4/1; weight/weight), self-assembly of nanoparticles occurs through electrostatic and non-ionic bonds. These nanoparticles can be lyophilized into dry powder and then re-formulated with aqueous solution (Figure 3A, Supplementary Figure 4). The lyophilized HKP (TGF-β1/COX-2siRNA) nanoplex powder once re-constituted has an average size about 150 nm in diameter and a Zeta potential about 40 mV; the nanoparticles are stable at 4°C, exhibiting potent silencing activity for TGF-β1 up to 12 months (data not shown).

To further understand the duration and distribution of the locally delivered siRNA, we used a human hypertrophic scar tissue implant mouse model. qRT-PCR analysis of the tissue samples from HKP (TGF-β1/COX-2siRNAS) treatments revealed TGF-β1 knockdown and COX-2 knockdown in a dose-dependent manner. For TGF-β1, the decrease was greater between 24–48 hours, whereas for COX-2, the decrease was greater between 48 and 96 hours post treatment (Figure 3B). Furthermore, two intrascar injections were conducted with one containing a naked Alexa Fluor labeled-siRNA and the other one containing the HKP packaged Alexa Fluor labeled-siRNA nanoparticle formulation. Tissue samples were collected at 4 hours, 24 hours and 48 hours post treatment injections, and analyzed under a Fluorescence microscope (Figure 3C). The naked siRNA is quickly dispersed after being injected into the scar tissue, and could not be detected after 24 hours. The HKP-packaged siRNA illustrated a quick dispersion and lasting release that can be detected even after 48 hours. Therefore, we predicted that HKP-packaged siRNA nanoparticle formulation represents a useful means for evaluation of the target gene silencing *in vivo*. When HKP-packaged TGF-β1/COX-2siRNAs combination was administrated through an intrascar injection, we observed potent silencing on days 1, 3 and 5 post treatment. This silencing activity resulted in parallel down regulation of both TGF-β1 and COX-2 with the greatest inhibition being seen at day 5 (Figure 3D). Examination of TGF-β1 and COX-2 mRNA by qRT-PCR showed a higher expression of both target genes in HS than in normal skin tissues (Figure 3E).

### HKP-packaged TGF-β1/COX-2 siRNA (STP705) reduces size of human hypertrophic scar

**Figure 4:**
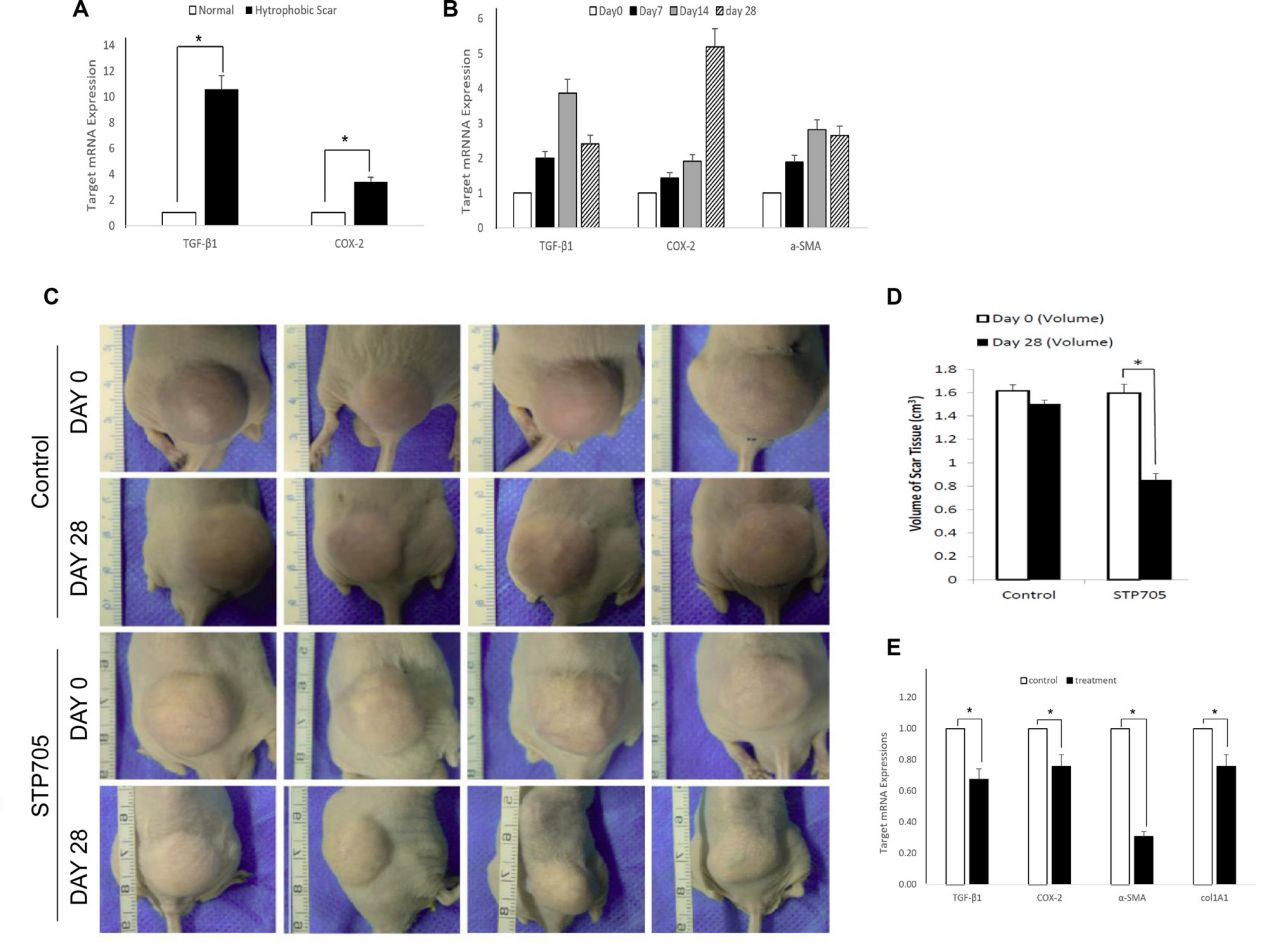
HKP (TGF-β1/COX-2siRNAS) Treatment Reduces Size of Human Hypertrophic Scar (**A**) TGF-β1 and COX-2 are highly overexpressed in Human Hypertrophic Scar. qRT-PCR results reveal significant upregulated expressions of TGF-β1 and COX-2 in human hypertrophic scar tissue (black bars), compared to those expressions in normal human skin tissue (open bars). *P < 0.05 (*N* = 4). (**B**) Expression dynamics of TGF-β1, COX-2 and a-SMA in human hypertrophic scar tissue after being implanted under mouse skin, at day 0 (open bar), day 7 (black bar), day 14 (grey bar) and day 28 (shade bar). *N* = 3. (**C**) Images of human hypertrophic scar implants, either treated with HKP (TGF-β1/COX-2siRNAS) or control aqueous solution, at day 0 and day 28th post treatments. (**D**) Quantitative illustration of the size changes of the human hypertrophic scar implants. The reduction of the scar tissue sizes is approximately 45% for HKP (TGF-β1/COX-2siRNAS) treated group (*N* = 4), *P < 0.05. (**E**) mRNA levels of TGF-β1, COX-2, a-SMA and Col1A1 mRNAs in the HKP (TGF-β1/COX-2siRNAS) treated scar implants were significantly down regulated at day 28 (*n* = 3). *P < 0.05.

As expected, we found that TGF-β1 and COX-2 were significantly over-expressed in human hypertrophic scar (HS) tissue from patients compared to normal skin tissue (Figure 4A). Human HS tissues were implanted onto nude mice subcutaneously for studying the pathophysiology of HS and the efficacy of siRNA silencing. After HS tissue was implanted, we analyzed these tissues at day 7, day 14 and day 28 post implantation. We then isolated mRNA from the tissue samples to determine the expression dynamics of TGF-β1, COX-2 and α-SMA using qRT-PCR analyses (Figure 4B). The expression of TGF-β1 and COX-2 in the implanted human scar tissues exhibited an unexpected pattern with a rapid increase of TGF-β1 versus a steady increase in COX-2. The TGF-β1 expression reached a peak level at day 14 while COX-2 expression was still up regulated on day 28 post implantation.

Based on the results from Figure 3D and Figure 4B, we initiated treatment on the implanted human hypertrophic scars on mice four weeks after surgery. A 20 μg/50 μl /cm3 HKP (TGF-β1/COX-2siRNAs) was administered to each scar implant using 5 aliquots into 5 different sites of the scar, with three repeated injections at 5 days intervals. The STP705 combination treated HS implants showed a significant reduction in size of implanted tissues at day 28 post-treatment (Figure 4C–4D), by about 45% compared to the untreated group. After tissue samples from those implants were further analyzed, we found that not only the targeted genes TGF-β1 and COX-2 were significantly silenced based on the qRT-PCR results, but other proteins such as α-SMA and col1A1 were also significantly down regulated (Figure 4E). This result provided strong evidence that the simultaneous inhibition of TGF-β1 and COX-2 enhanced scar reduction.

### STP705 reduces size of human skin grafts

**Figure 5:**
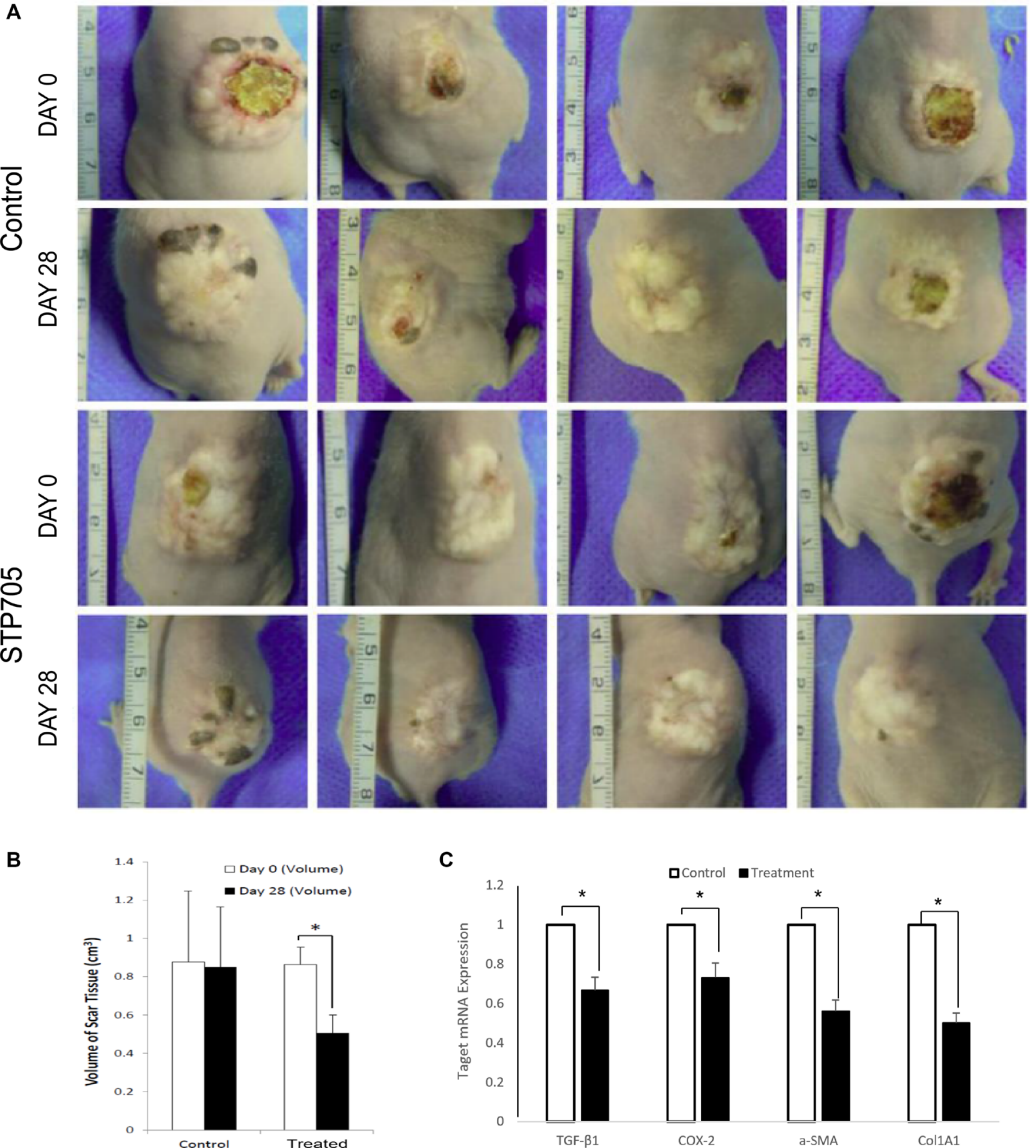
HKP (TGF-β1/COX-2siRNAS) Treatment Reduces Size of Human Skin Implants (**A**) Images of human skin implants, either treated with HKP (TGF-β1/COX-2siRNAS) or control aqueous solution, at day 0 and day 28th post treatments. (**B**) Quantitative illustration of the size changes of the human skin implants. The reduction of the skin tissue sizes is 38% for HKP (TGF-β1/COX-2siRNAS) treated group (*N* = 4), *P < 0.05. (**C**) mRNA levels of TGF-β1, COX-2, a-SMA and Col1A1 in the HKP (TGF-β1/COX-2siRNAS) treated skin implants were significantly down regulated at day 28 (*n* = 3). *P < 0.05.

Similar to human hypertrophic scar implants, human skin grafted onto the nude mouse is able to regenerate after being subjected to a full-thickness wound. This approach has been used to determine the cells involved in the connective tissue repair process following superficial wound injury. In addition, this model has been used to study the wound healing process of human skin. Whereas the hypertrophic scar model was established by transplanting hypertrophic scars with both macroscopic and histologic properties similar to human scars, this model ensures observing the entire process of hypertrophic scar formation. Thus it is ideal for studying hypertrophic scar [19]. The initial time and dosing regimens were similar to the treatment of the implanted human hypertrophic scars on mice. Four weeks after the surgery, 20 μg/50 μl/cm^3^ STP705 solution was injected into each skin graft using 5 aliquots to 5 different sites of the graft, with three repeated injections at 5 days intervals. The HKP (TGF-β1/COX-2siRNAs) combination treated human skin grafts resulted in a significant size reduction at day 28 post-treatment (Figure 5A–5B), by about 40% compared to the untreated group. After tissue samples from skin grafts were further analyzed, we found that not only the targeted genes, TGF-β1 and COX-2, were significantly silenced based on qRT-PCR analysis but that α-SMA and col1A1 were also significantly down regulated (Figure 5C). This result is similar to what we observed with the human hypertrophic scar implants and provides further evidence that simultaneous silencing of TGF-β1 and COX-2 enhances scar reduction.

### STP705 demonstrates a novel anti-fibrotic mechanism of action

**Figure 6:**
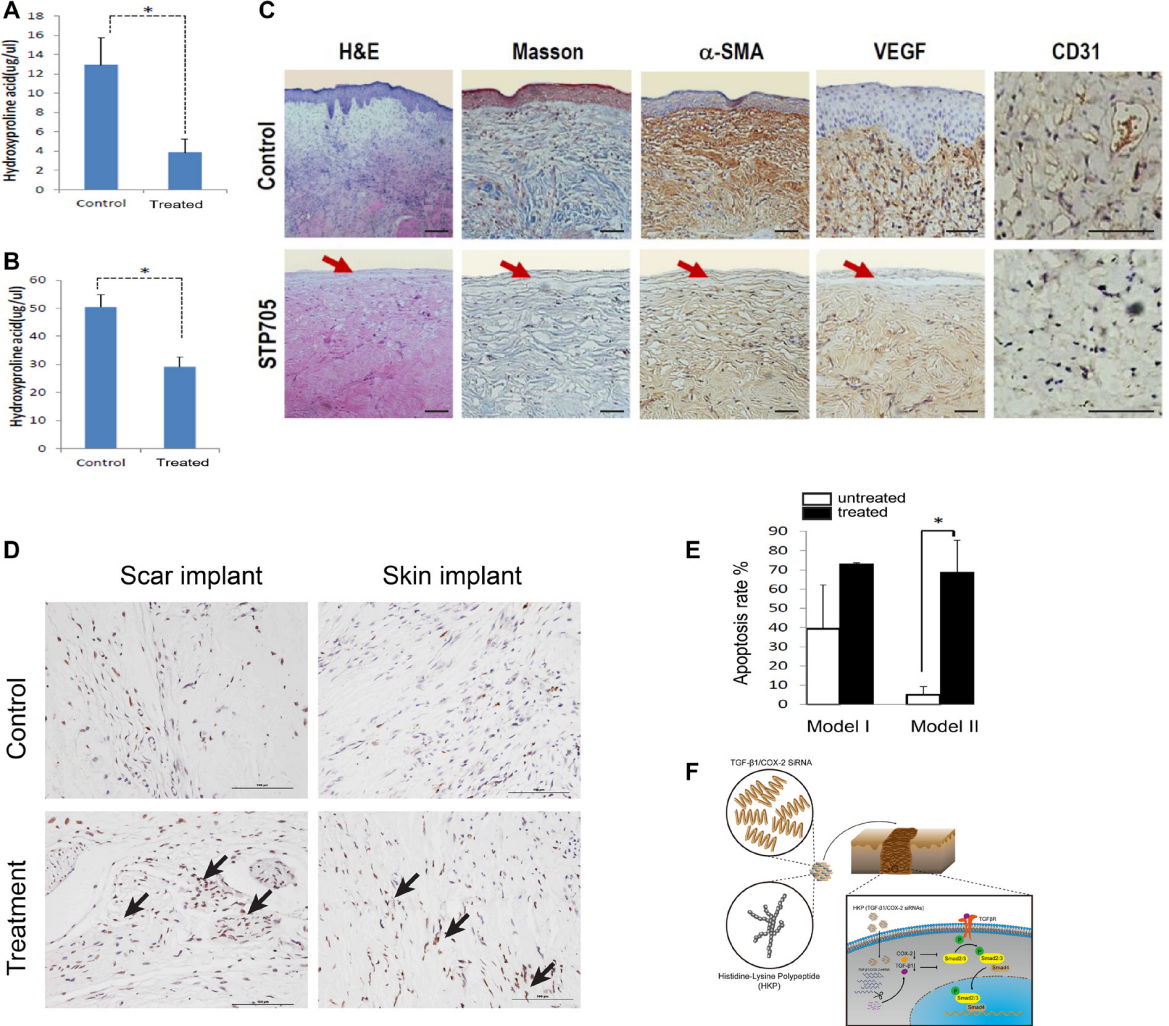
Anti-fibrotic Activity of HKP (TGF-β1/COX-2siRNAS) in Human Scar Tissues: Down regulations of hydroxyproline acid in human scar tissue implants (**A**) is about 70%, and (**B**) in human skin tissue implants is about 40%, *N* = 3, *P < 0.05. (**C**) Tissue samples with H&E and Masson's trichrome staining, and IHC staining with antibodies against human VEGF, CD31 and a-SMA proteins, revealed down regulations of the angiogenesis, micro vessel marker and fibrogenesis after repeated treatments with HKP (TGF-β1/COX-2siRNAS). Red arrows indicate epidermis layer of the skin. (**D**) HKP (TGF-β1/COX-2siRNAS) treatment induces fibroblast apoptosis in those implanted hypertrophic scar tissues and skin tissues (indicated by the arrows), and (**E**) quantitative measurements on the right. Model I and II refer to scar implant and skin grafting, respectively. (**F**) Diagram of the research.

To investigate the underlying biology of the observed scar tissue reductions with the human hypertrophic scar and human skin graft implants after STP705 treatment, we first measured hydroxyproline acid level from the tissue samples and then measured the differences between the treated and control groups. In both human scar and skin implants, STP705 treatment resulted in a significant down regulation of expression in hydroxyproline, compared to the control group (Figure 6A–6B). Furthermore, we compared treated (HKP -TGF-β1/COX-2siRNAs) and untreated tissues using H&E, or Masson's trichrome staining, or immunohistochemistry (IHC) staining against human α-SMA, VEGF and CD3. The marked differences of the tissue structures and expression levels of these pro-fibrotic factors were demonstrated (Figure 6C). We further measured the apoptotic activity of the fibroblasts *in vivo* as we did in the cell culture study, using a TUNEL assay. Histology images illustrated a marked increase in apoptosis of fibroblasts in the treated tissue samples (Figure 6D). Further quantitative analysis confirmed a significant increase of the numbers of apoptotic cells (Figure 6E).

## DISCUSSION

A dermal wound healing process can be classified into three phases: inflammation, cell proliferation and matrix remodeling, which involve multiple interactions within a complex network of pro-fibrotic and anti-fibrotic molecules [20]. After dermal injury occurs, the aggregated inflammatory cells become sources of growth factors and key enzymes of inflammatory cytokines such as COX2 are induced during the inflammatory phase. Fibroblasts are the most common cells in connective tissue and key players in skin wound healing process especially during the proliferation and remodeling phase, functioning to maintain the physical integrity of the connective tissue, participate in wound closure, and produce and remodel ECM [21]. When active angiogenesis and collagen synthesis ensue in concert with the tissue remodeling process, a delicate balance of deposition and degradation of fibroblast-expressed ECM determines normal skin wound healing or whether a wound heals but with HS. Regulation of fibroblast proliferation, their transition to myofibroblasts, and their apoptotic activity during the wound healing process can be critical modalities for therapeutic intervention.

Since TGF-β1 has been regarded as the critical factor regulating activation and proliferation of fibroblasts, inhibition of TGF-β1 is well accepted for targeted therapeutic approach against fibrotic scarring, and in fact, several candidate agents are currently in the clinical studies [22–26]. COX-2 and its major product, prostaglandin E2 (PGE2), are generally considered potent pro-inflammatory and proliferative mediators. Through a heterogeneous nuclear ribonucleoprotein A/B (HNRPAB), TGF-β1 coordinately up-regulates COX-2 expression in murine fibroblasts [27]. We also found that silencing TGF-β1 in human fibroblasts down regulates COX-2 expression, and vice versa (Figure 1C). Down-regulation of COX2 decreased the expression levels of SMAD2/3 and phosphorylated SMAD2/3, indicating that COX2 modulates the activation of TGF-β signaling pathway (Figures 2D, 6F). Although there are reports indicating that high levels of COX-2 transformed TGF-β1 into an anti-fibrotic regulator in cell culture studies, these observations may be due to the terminal stages of the cells used and may not reflect the early responses of TGF-β1 and COX-2 pathways to injury *in vivo* [12]. Moreover, evidence of increased expression of these two targets (TGF-β1 and COX-2) in the human hypertrophic scar tissue (Figure 3E) warranted our selection of these targets for therapeutic intervention. In addition, fetal wound healing studies in mice have demonstrated that reduced PGE2 and COX2 levels [13] resulted in lower collagen synthesis, suggesting that targeting COX-2 may be a potential therapeutic approach for limiting scar formation [14].

Recently, small interfering RNA targeting different signal molecules such as TERT [28], TGFBRI [29] and TIMP1 [30] demonstrated their ability to inhibit extracellular matrix deposition and the growth of fibroblasts in the scar. However, siRNA cannot readily penetrate the barriers of epidermis to reach fibroblasts in deep scar tissue. This transport barrier is a significant limiting factor to translate of RNA interference therapy in the clinical setting [31], and consequently, an efficient siRNA delivery *in vivo* is critical for the success of an siRNA-based drug. In our study, we evaluated the HKP-siRNA nanoparticle formulation through intradermal (intrascar) administrations, to determine whether a clinically viable siRNA therapeutic product can be realized for treatment of the skin hypertrophic scar [19, 32]. We have developed scalable procedures for HKP (siRNA) nanoparticle formulation, which not only facilitated efficient siRNA delivery but exhibited no signs of adverse and toxic effects. In comparison with two ongoing clinical studies using oligonucleotide inhibitors for the similar indications, EXC001 and RXI109, the therapeutic dose of STP705 formulation was at least 2 logs below what has been reported for these 2 other agents (data not shown). We believe that the enhanced potency of STP705 observed in this study comes not only from the dual-targeted drug design but also from HKP-enhanced delivery to the site of action (Figure 3B).

The therapeutic benefits observed in the human hypertrophic scar models of this further validates the benefit of simultaneous silencing of of two target genes. Using HKP-enhanced *in vivo* siRNA deliveries through intrascar injection, we observed significant silencing effects on TGF-β1 and COX-2 expressions by about 40% (Figures 4E, 5C), with only microgram level of the siRNA inhibitors. These results support higher transfection efficiency of HKP packaged siRNA nanoparticles than other vehicles mediated treatments [33]. Moreover, apoptosis of fibroblasts in hypertrophic scars [34] has been seen with various therapies in prior studies. In this research, the activation and upregulation of fibroblast apoptosis within human hypertrophic scars both *in vitro* (Figure 2A) and *in vivo* (Figure 6D–6E) after STP705 treatment revealed a novel underlying mechanism of action. The injection of HKP-packaged scrambled siRNA into the skin of normal mice showed no significant changes (data not shown). Additionally, cells treated with and without scrambled siRNAs gave similar results *in vitro*, suggesting that the effects of STP705 were not caused by non-specific gene silencing. Therefore, we hypothesize that simultaneously silencing of TGF-β1 and COX-2 within the human hypertrophic scars were able to inhibit inflammatory activity and proliferation of fibroblasts. This inhibition leads to reduced ECM deposition and enhanced collagen degradation which is consistent with studies in other fibrosis diseases [35]. These results indicate that STP705 is a clinically viable novel siRNA therapeutic to reduce human skin hypertrophic scarring.

The up regulation of fibroblast apoptosis in the cell culture and human HS and skin tissue implants confirmed the therapeutic potential of STP705 to avoid fibrotic scarring, by maintaining an optimized fibroblast proliferation and a balance between deposition and degradation of ECM production. Down regulation of α-SMA, Collagen 1, Collagen 3 and hydroxproline acid in both human fibroblasts and HS tissue, at both mRNA and protein levels after the treatments, implicates a complex network regulating skin fibrotic scarring. The results from this study have further advanced our understanding of the mechanism of actions of the pathophysiological pathways involved in the hypertrophic scar formation. The *in vivo* study showed a thinning of the epidermal layer in the STP705 treatment group, yet it is unclear whether this phenotype was caused by RNAi-mediated target gene knockdown or by the formulation. Further studies will be carried out to investigate the influence of STP705 on epidermis. Although side effects of STP705 need to be intensively studied, the synergistic activity of STP705 silencing both TGF-β1 and COX-2 at the proliferation stage and remodeling stage of skin wound healing process provides solid evidence that the skin hypertrophic scar formation can be potentially reversed through modulating the activity of fibroblasts within the scar.

## MATERIALS AND METHODS

### Sequence design and selection

25-mer blunt-ended siRNA duplexes targeting homologues regions of human, mouse and porcine TGF-β1 or Cox-2 mRNA sequences were designed (Table 1). Eight siRNA for each gene were screened in human PC3 cells. Briefly, 2.5×105 PC3 cells were seeded on the wells of 6-wells plates on the day before transfection in 2 ml of ATCC optimized medium supplemented with 10% of fetal bovine serum. On the next day cells were transfected with Lipofectamin2000 (Invitrogen, CA) packaged siRNA complexes according to the manufacture's recommendation. After 48-hours incubation, total RNAs were isolated from the cells with RNAqueous-4 PCR kit (Ambion, TX) followed by qRT-PCR analyses. Based on the potencies of target gene silencing of each siRNA sequence, the most potent siRNA was selected for future tests.

### Reagents

The siRNA oligonucleotides were purchased from either Qiagen (Valencia, CA) or Dharmacon (Boulder, CO). Histidine-lysine co-polymer (HKP) was provided by Biopolymer lab at the University of Maryland or purchased from Ambio Pharma Inc. (Shanghai, China). Primers PCR were purchased from Qiagen (Germantown, MD) or Genepharm (Suzhou, China). Rabbit polyclonal anti-alpha smooth muscle Actin (α-SMA) antibody, Rabbit polyclonal anti-COX2 antibody, Rabbit monoclonal anti-TGF-β1, Rabbit Monoclonal anti-human MHC class 1 antibody, Rabbit polyclonal antibody to CD31, and Rabbit polyclonal antibody to VEGF were purchased from Abcam Corporation (Iowa, USA). Rabbit monoclonal anti-SMAD2/3, anti-p-SMAD2/3 and anti PAI-1 antibody were purchased from Cell Signaling Technology (Danvers, MA), The Bio-Rad iScript reverse transcription kit and IQ Sybr Green Supermix reagents (Hercules, CA) were used for qRT-PCR performed with Bio-Rad MyiQ Thermal Cycler. COX2 inhibitor Celecoxib (Catalog No.S1261) and TGFβ receptor I (TBR1) antagonist Galunisertib (LY2157299) (Catalog No.S2230) were purchased from Selleck Chemicals (Shanghai, China).

### Cell culture

Human PC3 cells (prostate adenocarcinoma) was purchased from the ATCC (Manassas, VA) and cultured in ATCC-formulated F-12K medium with 10% fetal bovine serum (FBS). HS tissues were cut into small pieces of 0.5–1 mm^3^. and incubated for 2–4 hours with 0.25% type I collagenase until tissue became flocculent. Following incubation cells were pressed through a sterile nylon mesh, washed with PBS and cultured in DMEM medium with 20% FBS, 100 U/ml penicillin and 100 μg/ml streptomycin with incubation at 37°C in 5% CO2. This study was conducted according to the principles expressed in the Declaration of Helsinki. All participants in this study or their parents or legal guardians provided written informed consent for the collection of samples and subsequent analyses. Samples used in this study were listed in Table 2. The growth medium was changed every 2–3 days and only low passage cultures (between passage 3 and passage 6) were analyzed for further experiments. SiRNA or pcDNA3.1 plasmids transfected cells were collected after 48 hours, if not otherwise stated. Inhibitors of TBR1 and COX2 were added at a concentration of 100 nmol/L and cells were incubated for 48 h before collected.

### Growth inhibition *in vitro*

Cells were seeded into the wells of 6-well plate at density 106 cells per well in 3 ml media. Six hours later, the media was replaced with fresh media containing HKP alone, HKP (TGF-β1/COX-2siRNAs), HKP (TGF-β1siRNA), or HKP (COX-2siRNA) at concentrations of 10 ug/ml HKP loaded with 5ug/ml siRNA. For the Annexin V/PI assay, transfected fibroblasts were harvested and resuspended at a density 106 cells/ml and then washed with cold PBS twice. Cells were resuspended in 60 μl of binding buffer, and Annexin-V-FITC (4 ul; 20 ug/ml) (Dead Cell Apoptosis Kit with Annexin V Alexa Fluor**^®^** 488 & Propidium Iodide, Invitrogen, CA) was added. Cells were incubated in dark on ice for 15 minutes. Before processing, PI (4ul; 50ug/ml) was added to the suspension. For cell cycle analysis, the transfected fibroblasts were harvested and washed with PBS three times. Cells were fixed with 70% ethanol at 4°C overnight and then washed twice with PBS. Cell pellets were resuspended in propidium iodide (500 μl; 50 μg/ml) and kept at 4°C overnight. The suspension was run through a single cell sieve to make a single cell suspension, which was analyzed by FACS (EPICS Altra, BACKMAN COUNLTER, Holland).

### Real-Time qPCR

Total RNA was extracted from cells and tissues using Trizol Reagent (Invitrogen). Reverse transcription was performed using PrimeScript RT-PCR Kit (Takara Bio) according to the manufacture's protocols. Real-time quantitative Reverse Transcription PCR was performed using the SYBR Select Master Mix (Applied Biosystems) and an ABI 7500 real-time PCR system (Applied Biosystems). Primers used for Real-Time qPCR were listed in Table 3.

**Table 3:** Primers sequences used in the study for qRT-PCR analyses

	Forward	Reverse
**hβ-actin**	5′-ACCAACTGGGACGACATGGAGAAA-3′	5′-TAGCACAGCCTGGATAGCAACGTA-3′
**hTGFβ1**	5′-GAGCCTGAGGCCGACTACTA-3′	5′-CGGAGCTCTGATGTGTTGAA-3′
**hCOX-2**	5′-ATTCCCTTCCTTCGAAATGC-3′	5′-GGGGATCAGGGATGAACTTT-3′
**hα-SMA**	5′-GGCATTCACGAGACCACCTAC-3′	5′-GGGGCGATGATCTTGATCTT-3′
**hCOL1A1**	5′-AGGGCCAAGACGAAGACATC-3′	5′-GTCGGTGGGTGACTCTGAGC-3′
**hCOL3A1**	5′-TGAAGGGCAGGGAACAACT-3′	5′-GGATGAAGCAGAGCGAGAAG-3′

### Western blotting analysis

Total cell lysates were prepared by solubilization in RIPA buffer containing protease and phosphatase inhibitors (Selleck Chemicals). Protein concentration was measured by the Bradford method. After 4%–20% SDS–PAGE, proteins were transferred to 0.45 um PVDF membranes (Millipore), blocked in 0.5% skim milk for 1 h at room temperature, incubated overnight with the primary antibody and secondary antibody for 1 h, with three washes of 10 min in TBS plus 0.1% Tween-20 after each incubation. The proteins recognized by the specific antibodies were visualized by chemiluminescence methods (High-sig ECL Western Blotting Substrate, Tanon, China) using peroxidase-conjugated anti-rabbit secondary antibodies as mentioned above. Densitometric quantification was performed in unsaturated images using ImageJ (NIH).

### Plasmid construction

The cDNA encoding the *COX2* ORF was amplified by PCR using primers incorporating restriction enzyme sites (Hind III and XbaI) from NCBI online. The PCR fragment was cloned into the digested plasmids of pcDNA3.1 leading to the production of pcDNA3.1-*COX2*, with the following primers: *COX2*-sense: 5′- TATAAGCTTCCCTCAGACAGC AAAGCCTA-3′ and *COX2*-antisense: 5′- CTAGTCTAGA CTACAGTTCAGTCGAACGTTCTTTTAG-3′. Plasmids were isolated and purified using anion exchange columns (QIAGEN, Hilden, Germany) and all constructs were sequenced. Cells were transfected using Lipofectamine2000 reagent (Invitrogen) according to the protocol from the manufacturer.

### Detections of α-SMA and hydroxyproline acid

The cells were fixed with 4% paraformaldehyde for 10 minutes at room temperature and washed three times with PBS. Re-naturalization solution was added drop-wise to designated area encircled by an indelible pen and incubated for 5 minutes. Subsequently, cells were incubated for 30 min in the permeabilization solution (PBS, 0.1% Triton X-100), washed with PBS three times and blocked with 10% goat serum for 1 hour. Cells were then incubated with anti-α-SMA antibody (1:100 dilution) at 4°C in a moisture box overnight. Cells stained with goat-anti-rabbit secondary antibody (1:200) at room temperature in dark for 30 minutes and counter-stained with DAPI at room temperature for 1 minute, washed with PBS and observed under a fluorescent microscope. Hydroxyproline Colorimetric Assay Kit (BioVision, USA) was used for detection of hydroxyproline accordingly to the manufacturer's instruction.

### Nanoparticle preparation

optimized Histidine-Lysine polymers (HKP) were used for siRNA delivery *in vivo*. One of HKPpeptides, H3K4b, having a lysine core with four branches that contain multiple repeats of histidines and lysines, was used for packaging siRNAs targeting TGF-β1 and COX-2. The HKP carrier: siRNA payload ratio was 4:1 by mass. The HKP and siRNA self-assembled into nanoparticles with an average size of 150 nm in diameter. These HKP (siRNA) nanoparticles suspended in an aqueous solution were injected into the scar tissue.

### SEM and TEM analyses

A scanning electron microscopy was conducted when HKP (siRNA) nanoparticles were packaged as mentioned above. Samples for SEM were prepared by diluting above nanoparticle solutions to 0.1 mg/ml. Diluted samples were then dripped onto silicon wafers and air dried. The samples were then sputter-coated with a 2nm layer of a palladium /gold alloy. Pictures of nanoparticles were acquired under SEM (Hitach S-480, Japan) at 20°C, 60 RH (Figure 3A–3B). A transmission electron microscopy (TEM) was conducted when the transfected cells were fixed with 2% osmic acid for one hour. Cells were dislodged with a rubber scratcher and collected by centrifugation. Cell pellets were sectioned and analyzed under TEM. TEM images were taken with a CM-120 Bio Twio (PHILIPS, Holland) (Supplementary Figure 1).

### Mice

The 8-week old male nude mice (nu/nu Balb/c) were purchased from Center for Experimental Animals in Shanghai, China. Animal housing and experiment protocols were approved by the Independent Ethics Committee of Shanghai Ninth People's Hospital, Shanghai Jiao Tong University School of Medicine.

### Human hypertrophic scar tissue implant model

Skin hypertrophic scar tissue was obtained from a surgical excision with the informed consent and was trimmed off subcutaneous fat and cut into pieces of 2 cm^2^. 6–8 weeks old male nude mouse was anesthetized with 10% chloral hydrate and a piece of trimmed hypertrophic scar tissue was implanted under the skin on the mouse back. Scar tissue was fixed to the mouse deep fascia with 4–5 sutures before skin was closed.

### Human skin tissue grafting model

The tissue samples used in experiments were from skin excisions of three women (ages 23–36) that had undergone breast reconstruction for treatment of macromastia. All patients had signed the informed consent voluntarily. Skin excisions were acquired in sterile condition, cut into size of 2 cm × 1.6 cm and kept in 20% FBS DMEM media at 4°C before use. Male nude mice of 6–8 weeks old were anesthetized with 10% chloral hydrate and a piece of skin (2 cm × 1.6 cm) was excised from the back. A same size human skin was grafted to replace the excision by sutures to subcutaneous fascia and surrounding mouse skin. A stack of sterile cotton was placed on the top of the graft and tightly wrapped. Stitches were taken off two weeks later.

### Quantification of therapeutic effects *in vivo*

Four weeks after the human hypertrophic scar implantation, 20 ug/50 μl/cm^3^ of HKP (TGF-β1/COX-2siRNAs) was injected into the scar established on the mouse body. To ensure even drug distributions, injections were performed into 5 areas - 4 quadrants and 1 center. Every dose of the drug was injected in 5 equal aliquots. Scars were injected three times for the period of 15 days (once per 5 day). Scar size was evaluated before and after treatment. At day 1, 2, 5 and at the end of the experiment animals were euthanized, and scar tissue was immediately harvested and homogenized in Trizol solution with Polytrone (Brinkmann Homogenizer Polytron PT 10/35). Total RNA was extracted and RNA level of TGF-β1, COX-2, α-SMA, Col1a1, and Col3a1 was analyzed by qRT-PCR. The *in situ* Cell Death Detection Kit from Roche (South San Francisco, CA, USA) was used for detection of apoptotic cells from the STP705 treated scar tissues, following the vendor's instruction.

### Statistical analysis

Mean ± SD was used for cell culture results, and mean ± SE was used for *in vivo* results. Student's *t*- test was used to determine significance between two groups. *P*- values < 0.05 were considered statistically significant. IBM SPSS statistics ver. 20 was used for statistical analysis.

## SUPPLEMENTARY MATERIALS FIGURES



## Abbreviations

siRNA: small interfering RNA
HKP: Histidine-Lysine Polymer
HS: Hypertrophic Scar
HSFs: Hypertrophic Scar fibroblasts
ECM: extra cellular matrix
TGF-β1: transforming growth factor-β1
COX-2: cyclooxygenase-2
TBR1: TGF-β receptor I.
