# Supplementary material for: Simultaneous silencing of TGF-β1 and COX-2 reduces human skin hypertrophic scar through activation of fibroblast apoptosis
Source: Oncotarget. 2017 Sep 14;8(46):80651–65. doi: 10.18632/oncotarget.20869 (PMC5655228; doi:10.18632/oncotarget.20869)
Supplement: Supplementary file 1 [file oncotarget-08-80651-s001.pdf]

Simultaneous silencing of TGF-β1 and COX-2 reduces human skin hypertrophic scar through activation of fibroblast apoptosis

SUPPLEMENTARY MATERIALS

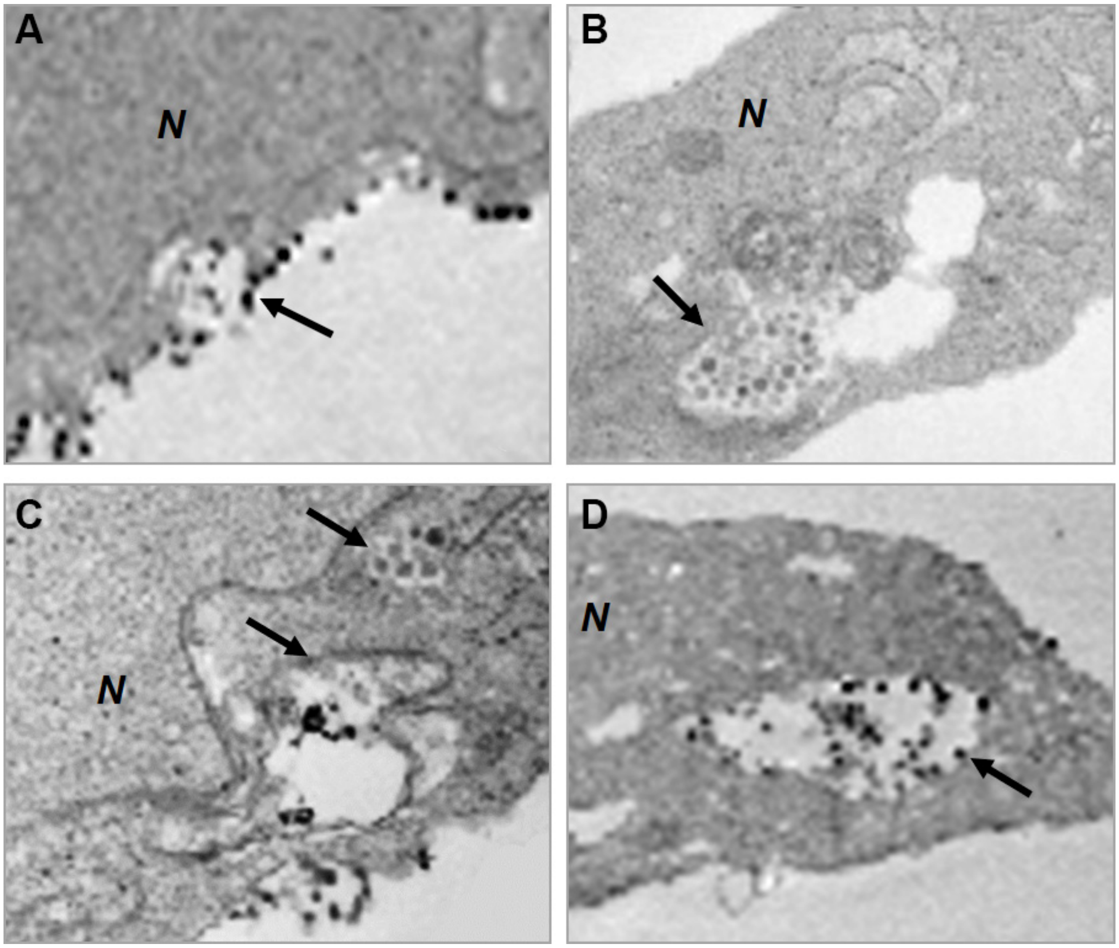

Supplementary Figure 1: TEM images of cellular uptake of siRNA. N indicates the cell nucleus and the arrows identifies the HKP/siRNA particles. (**A**) HKP/siRNA nanoparticles attached to the cell membrane; (**B**) HKP/siRNA nanoparticles are entering human fibroblasts via endocytosis; (**C**) The HKP/siRNA nanoparticles movements in the cytoplasm having endosome escape; and (**D**) HKP/siRNA nanoparticle enhanced fibroblasts apoptosis.

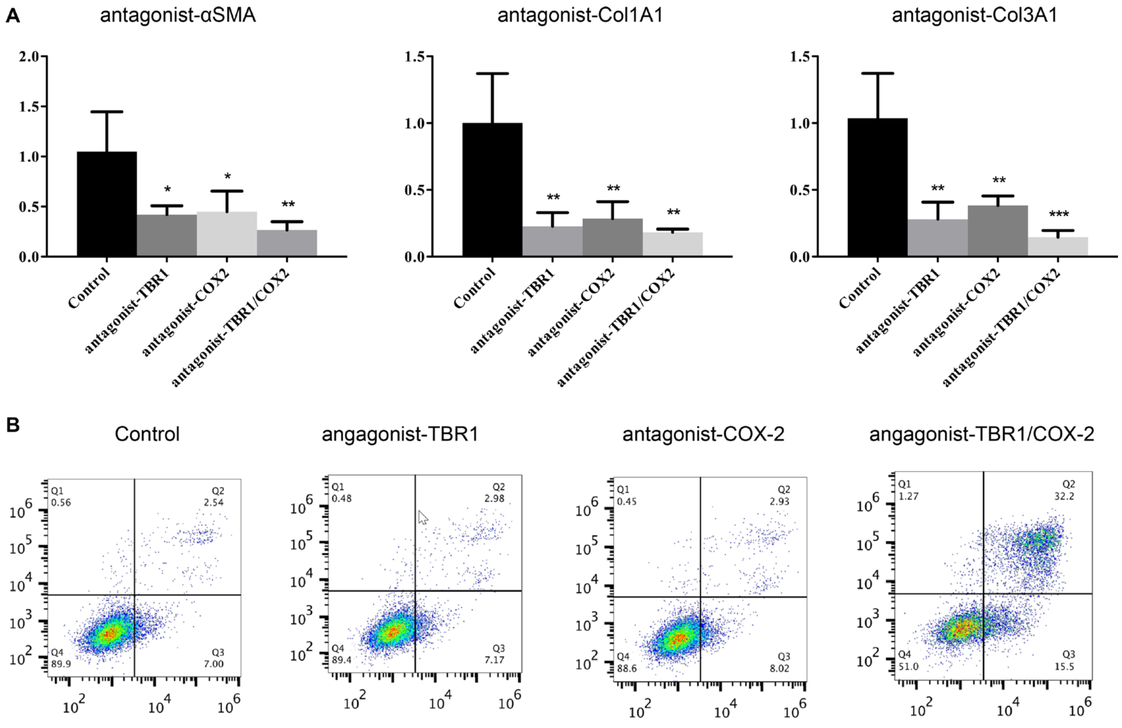

Supplementary Figure 2: Cells treated with COX2 inhibitor Celecoxib or TGFβ receptor I (TBR1) antagonist Galunisertib, or in combination, showed (**A**) a decreased mRNA expression level of α-SMA, COL1A1 and COL3A1. (**B**) Apoptotic activity was enhanced when HSFs were treated with inhibitors of both TBR1 and COX2. HSFs incubated with DMSO were taken as control.

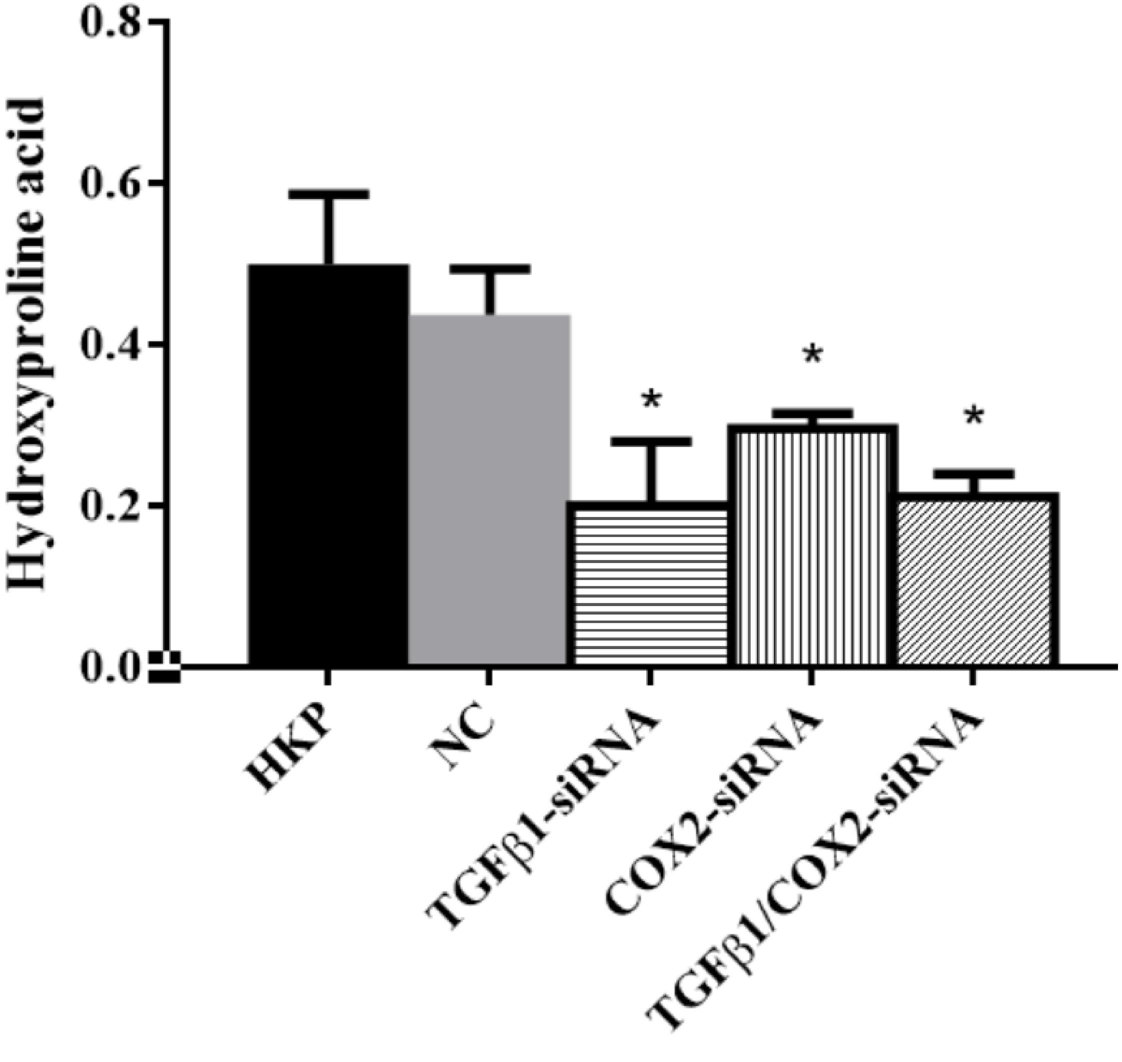

Supplementary Figure 3: Hydroxyproline acid activity within the cells was significantly down regulated with TGF-β1siRNA, or COX-2siRNA alone, or in combination. $N = 4$, * $P < 0.05$.

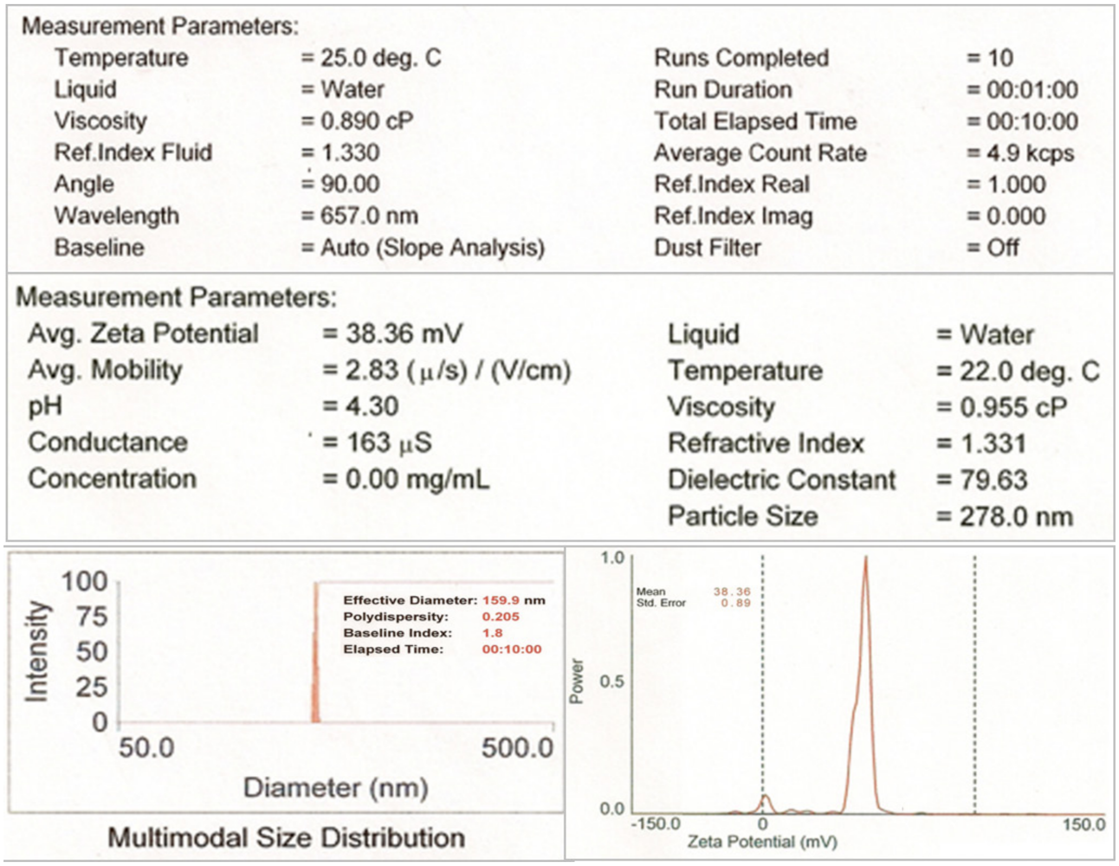

Supplementary Figure 4: Physicochemical properties of HKP (siRNA) nanoparticles. Particle sizes (upper panel and lower left) and zeta-potentials of HKP (siRNA) nanoparticles (middle panel and lower right) were measured, resulting in an average particles size of about 150 nm in diameter and a zeta-potentials of 38 mV.
